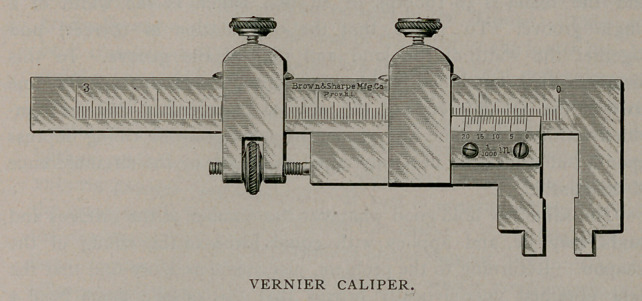# The Missile and the Weapon2Read at the thirty-second annual meeting of the Medical Association of Central New York, at Syracuse, October 17, 1899.

**Published:** 1900-05

**Authors:** A. L. Hall

**Affiliations:** Fairhaven, N. Y.


### THE MISSILE AND THE WEAPON.[2]

BY A. L. HALL, M. D., Fairhaven, N. Y.

THE missile and the weapon having the widest use in civil life, and consequently the greatest medico-legal interest, are the conoidal bullet and the pistol. For this reason, they have been selected for a few remarks concerning what may be determined from a careful study of them. First, we will consider the missile and what may be

1. See table of eye-terms on a preceding page.

2. Read at the thirty-second annual meeting of the Medical Association of Central New York, at Syracuse, October 17, 1899.

learned from it: Inspection may show tissue and blood stain, filaments of clothing, hair, lubricant, grains of unconsumed powder, dirt and so forth, clinging to its surface. These should be removed, examined microscopically and preserved for future use. After being deprived of foreign matter, the bullet should be accurately weighed, its contour noted and the effect of impact observed. Every marking should be carefully studied. Those originally present and due to manufacture, should be differentiated from those produced by bruising, as well as those caused by the rifling of the weapon. Then the bullet should be carefully measured and its length and various diameters obtained in thousandths of an inch. This will require the use of a caliper micrometer—such as now shown to you—which is a highly accurate instrument, and is manufactured by the Brown & Sharpe Manufacturing Company, of Providence, Rhode Island. A

Caliper Micrometer.

little patience, with some study, will easily make one competent to skillfully measure in thousandths of an inch, such measurements as are ordinarily made with this instrument.

The form, weight and caliper of the missile must necessarily be depended upon to obtain its caliber. And right here, I desire to say a word about the term caliber. As commonly understood, it means the diameter of the missile and that of the bore of the weapon. Thus the various calibers are known as twenty-two, thirty-two, thirty-eight and so forth. It being intended by these designations to convey the idea that these calibers are really so many hundredths of an inch. This is only a convenient approximation. Such a thing as an actual 22-caliber bullet, or weapon, measuring 22-100ths of an inch, is difficult, or impossible to find, except occasionally in rim-fire

HALL—THE MISSILE AND THE WEAPON.

cartridges. And what has been said respecting the 22-calibers is equally true of the other calibers. A 38-calibered Smith and Wesson pistol measures only 350-1000ths of an inch and a properly fitting bullet measures 360-1000ths of an inch. A 38 Colt's army revolver has a bore of 363-1000ths of an inch and its bullet calipers only 350-1000ths of an inch. In the Smith and Wesson pistol the bullet is larger than the bore of the weapon, and in the case of the Colt's, it is smaller than the bore and easily drops through it. The reason for these differences, I will not stop to explain, but will pertinently remind you, that if a fairly-well preserved bullet is found having a diameter of 360-1000ths of an inch, or a close approximation thereto, you may safely conclude that you have a 38-caliber missile. And if there are distinct rifling marks upon it, you may be certain that it came from a 38-calibered weapon.

Having determined the caliber, the further identification of the missile may be undertaken. For this purpose nothing is more important than the discovery of the original markings, or the so-called "details" of the bullet, which have been produced during the process of manufacture. The principal markings are known as the canneluring, knurling and cupping, which I will briefly explain. (See plate, Figs. 1 and 2.) Usually, bullets have one or more grooves encircling the body, either within or without the shell of the cartridge for holding the lubricant, known as the cannelure. In most of instances, the cannelure has transverse markings resembling the milling upon the edge of coins, or the marking of the bark of a tree may be imitated. These markings are technically known as knurls. The cupping is the basal depression which is usually present in most of bullets. The cannelure with its knurls and the cupping should be carefully examined. If lubricant is present it should be removed and its composition determined. The form, width and depth of the cannelure should be noted, and the number and direction of its knurls, together with any special peculiarities should be observed. The deciphering of these markings is a slow process and requires considerable toil and patience, but the result will, when carefully worked out, not only reward the investigator, but he will be actually surprised at the value of his findings.

In a good many bullets the knurling may be faulty and in part broken, or a portion absent. I have observed this in a large series of bullets of the same manufacture, which was occasioned by faulty machinery. This instead of being an obstacle to identification, may prove a strong aid. The importance of this is better comprehended, when it is known that bullets from the various manufacturing concerns throughout the country, when made in accordance with the specifications furnished by the weapon manufacturer, vary widely in the minute details which go to make up the cannelure, knurling and cupping. A study of the bullets, as found in the fixed ammunition offered for sale, will satisfy you that this is true.

In the study of the bullet details, or markings, a good Coddington lens is a desirable help, and many times the low powers of a microscope may be advantageously employed. In making measurements upon bullets, never use an eye-piece, or stage micrometer for correct results will seldom be obtained. Use one of the micrometers previously mentioned, or if measuring the lands made upon the bullet by the rifling of the weapon, employ a Vernier caliper.

The cupping of a bullet serves as a valuable guide to identification, and experts from the manufactories depend largely upon this as an aid to recognition. The reason for this is found in the fact that the base of a bullet is the least liable to injury and deformity, hence the cupping may be preserved when the cannelure and knurling have been destroyed by the force of impact. This is especially true when the missile strikes the petrous portion of the temporal bone. The cupping may act as a receptacle and contain portions of lubricant, unburnt powder, powder smut, and so forth, and in the case of bullets, which have long been in contact with black powder, considerable oxidation will generally be observed within the cupping and upon the basal end of the bullet. Usually the finding of such material is regarded as insignificant, but nevertheless, circumstances may arise whereby it may become important.

Manufacturing experts pay but little attention to the size and depth of the cannelure and the number and direction of the knurls. Therefore, if there be nothing distinctive about the base of the bullet, they are liable to fail to identify missiles which they have manufactured. This I have known to occur with well informed manufacturing experts, when the cannelure and knurls retained sufficient distinctive markings for a positive identification.

Then, there are many bullets which have become obsolete and their source of manufacture unknown. Or the bullet may have been made outside of a manufactory and its origin thus become a matter of speculation. In such cases, and in other instances where unusual disfigurement exists, the knowledge of a person specially skilled in the recognition of disfigured bullets may be utilised with advantage.

The final step in the examination of the missile is the consideration of the weapon markings. Usually, if the bullet has been fired from a rifled weapon, the rifling marks will be easily seen. These markings appear as alternate ridges and depressions extending longitudinally from the base of the bullet. When free from disfigurement, they are definite and regular in outline. The ridges are called *lands* and the spaces between the lands are known as *grooves*. At this point, it is well to explain to those who are uninformed in matters of this kind, that the rifling impression upon the bullet is the exact reverse of the weapon rifling. The land upon the bullet being produced by the groove of the weapon rifling, and the groove upon the missile by the land of the weapon. The lands of the bullet should equal in width and thickness that of the depth and width of the grooves of the weapon, while the lands of the weapon equal in width and thickness that of the depth and width of the grooves found upon the missile. The number of lands always equals the number of the

VERNIER CALIPER.

grooves. Sometimes the lands and grooves are each of like width and depth. Usually the lands are not of the same width as the grooves. Decided variations will be found in the lands and grooves of different styles of weapons, although they may be of the same caliber. Consequently, the same variations will be impressed upon the bullet, and these differences must be given careful consideration.

Having found rifling marks upon the missile, count them and measure their width by means of a Vernier caliper, such as illustrated above. The use of this instrument requires considerable skill to obtain correct results. To illustrate the method for making correct measurements and accurate computations, I offer the following problem for your consideration: Given a fired bullet, such as shown in Fig. 3, "mushroomed" by impact, but with well preserved body and base, presenting distinct rifling impressions upon its circumference and having a series of diameter measurements closely approximating 363-1000ths of an inch. Taking 363-1000ths of an inch and multiplying it by the well-known ratio number 3.1416, the result is 1140+thousandths of an inch, which is the circumference of the bullet. There are six lands and the same number of grooves impressed upon the surface of the bullet. You observe that the lands are several times wider than the grooves. With the Vernier caliper carefully measure the lands at as many points as possible until verified measurements are obtained. Assume the measurements to show the breadth of the land to equal 160-1000ths of an inch. Six times 160-1000ths inch equals 960-1000ths inch, which is the combined width of the lands. Taking 960-1000ths of an inch from the bullet circumference—1140-1000ths inch and there remains 180-1000ths of an inch of the bullet's circumference, which must be covered by the grooves. Dividing this fraction by 6, the number of the grooves, and the result is 30-1000ths of an inch, which is the width of a single groove. To prove that the computation is correct, add together the width of the land and that of the groove. In this instance, their combined width is 190-1000ths of an inch, or one sixth of the circumference of the missile. Remember that the combined width of the land and groove in a six-grooved rifling impress upon a bullet must of necessity equal one-sixth of the circumference of the missile.

The same law hold good whatever the number of the grooves and lands may be, and applies with equal force to the rifling of the weapon. Returning to the problem, just considered, we find that the data obtained unequivocally proves that the missile came from a 38-caliber weapon whose bore, lands and grooves measure respectively 363-1000ths, 30-1000ths and 160-1000ths of an inch. With these facts to aid us, the particular kind of weapon may be determined with certainty. There are other things about the missile which have not been considered, but will be mentioned in connection with the weapon.

### THE WEAPON AND WHAT MAY BE LEARNED FROM IT.

Ordinarily, many important and interesting circumstances attend the finding of a weapon which has been used to commit a crime. These, I will not mention, but will confine my remarks strictly to things which pertain to the weapon itself, making especial reference to those which serve to connect it with the missile.

A careful inspection of the weapon should be made and external staining, whether made by finger-marks, powder smut, blood and other foreign matters, should be noted and their exact nature determined. The number of the weapon, its caliber, make, length of barrel, weight and general condition should be recorded. The next point to consider is whether the weapon has, or has not been recently discharged. If recently fired, the bore of the weapon will be covered; if black powder has been used, with a dark, semi-liquid, greasy substance, which adheres to the finger and leaves a dark stain. In addition to these signs, there will be a strong odor of sulphuretted hydrogen, which may be easily detected during the first day, and usually for two or three days longer. Exceptionally, the odor may be detected for a period of seven to ten days. The weapon may be placed in some close receptacle, or covering, immediately after firing and by these means the odor may be retained for a long period of time. The color of the stain within the barrel, for the first twelve hours, undergoes no appreciable alteration, but at the end of the first day small points of color change will be noticed in the deposit. This change gradually extends and by the end of the third or the fourth day, it has been completed and the deposit has assumed a light gray color without staining properties. This change is known as the "efflorescence" and is due to the crystallisation of the products of fired gunpowder. Under the microscope, the efflorescent material is resolved into numberless white crystals with an admixture of minute particles of unburnt powder and charcoal. If rust is present within the barrel, the crystals may be reddened from this cause. For a few days, after the completion of the crystallisation of the deposit, the interior of the barrel presents a frost-like appearance which continues until the efflorescence becomes broken down by the further chemical changes due to oxidation. Generally by the end of the first month, and sometimes before, the deposit, to the unaided eye, has lost its bristling crystalline appearance. It has become smoother through oxidation, and if not affected by weapon rust, its color is slightly lightened. When this stage has been reached, the deposit for succeeding months undergoes little, if any, appreciable change, provided moisture is absent. The microscope shows the crystals at this stage the same as during the period of completed efflorescence, and the only appreciable differences will be their increased clearness, occasioned by the casting off of the blackened products of powder combustion, which like the crystals have clustered together by themselves.

The product of black gunpowder combustion has an alkaline reaction. In a large series of carefully conducted tests, I have never failed to obtain a marked bluing of reddened litmus paper, when moistened and brought in contact with these deposits. It is a constant peculiarity and always present, and it matters not whether the test be made immediately after firing, or if months and even years have elapsed, alkalinity is detectable, if any deposit is found within the barrel of the fire-arm. I show you an old pistol barrel, originally a part of a 22-caliber Colt's revolver which has been in my possession for more than two years. It is badly rusted, yet its interior shows, at this time, pronounced alkalinity, due to the presence of gunpowder residuum. When given to me, the ends of the barrel were filled with earthy matter and the outside was covered with fresh earth, it having been exhumed from a flower bed where it had been buried for a year. I have credible information that it has not been fired for fully fourteen years, it having been in its present broken condition during that time, and there are facts connected with its history, which render it probable that it has not been fired during the past twenty or more years. Shortly after I received this barrel, I made a test which showed it to be alkaline, and I also made a microscopic examination of the encrustation found upon the walls of its bore. These possessed the ordinary characteristics of fired gunpowder, such as have been previously described. Clear crystals, crystals discolored by the action of iron, charcoal and particles of unconsumed powder were there in abundance.

I shall not say anything concerning the chemical analysis of the deposit found within the barrel of a weapon, for the reason that the character of these deposits, as shown by microscopic examination and the litmus test, are as conclusive as any that may be obtained by chemical process.

If the weapon is a revolver, examine the cylinder and its chambers. Note evidences of firing, such as powder staining, the presence of fired shells, their number and kind, with marks of manufacture and all of their smaller details. Caliper the shells and bullets, if any are found. Weigh each bullet and the powder in each shell. Inspect the bullets carefully for peculiarities. Record the width and depth of the cupping and of the cannelures, together with the number, direction and peculiarities of the knurls. Compare these details with those found upon the fired bullet, as a means of identification, and for the purpose of showing that the fired bullet is like those found in the weapon. Take the measurements of the bore of the weapon and the diameter of its cylinder chambers, and compare these with the bullets and shells found. Inspect the rifling and note its peculiarities, and whether there has been any alteration or defacement due to rust, or injury. Sometimes the end of the barrel may become battered, and defacement of the rifling result in such a way, that a discharged bullet, will show markings which have been caused by these injuries. Then the sights should be examined and their mode of fastening should be observed. .I have known of an instance where bullet defacement resulted from the sight being driven through the rifling with the effect of invariably producing a noticeable tear upon the surface of a bullet when fired. In making an inspection of a weapon, evidence of this character should never be overlooked.

We next come to the consideration of the weapon rifling. This should be minutely inspected and the number of its lands and grooves noted. What I have previously said concerning the impress of the rifling upon the missile should be remembered. The measurement of the lands and grooves of the rifling should be taken. The following method will give accurate results: Carefully clean the rifling and lubricate with a good oil. Select a well-fitting bullet, freed from dirt and lubricant, and place it within the barrel at a distance of from one to two inches from the muzzle. Place behind it, within the barrel, a closely fitting drill rod of sufficient length. Above it place a similar piece of rod. Now rest the farther end of the lower rod against a firm support, and by a few smart blows with a hammer to the outer end of the upper rod, firmly compress the lead until it has snugly impressed itself into the grooves of the rifling. Remove the upper rod, and by a few light blows to the lower rod, expel the lead core and measure its lands with the Vernier caliper. Then by computation obtain the width of the grooves. Verify the measurements by those obtained from several plugs compressed within the rifling. Compare these measurements with those found upon the missile, and if they agree, you may be sure that the bullet was fired from this weapon, or one like it. Again, a bullet similar to the fired bullet may be lightly compressed within the rifling in imitation of a fired bullet, or a fired bullet may be recovered, in a nearly perfect condition, by firing into a bag of meal. The impressions thus obtained may be measured and compared with the lead core and the original bullet.

Photographs 4 and 5 show enlarged bullets which have been lightly compressed within the rifling. Figure 4, shows two bullets of like form, weight and caliber compressed within the same rifling. The number of the knurls and their direction differ and the cannelur-ing is unlike. Figure 5, shows two bullets which are precisely alike in all of their details. One has been compressed within a rifling having narrow grooves, while the other has been similarly treated in a weapon of like caliber, but having a much wider groove. A little study of these figures will, I believe, convince you of their value as means of identification.

Figure 6 represents two cartridges of like caliber, with shells exactly alike, made by the same manufacturing concern, containing bullets similar in general form, weight and composition, and which, by the manufacturers, were supposed to have no differences. Inspection shows a wide variation in the width and depth of the cannelures, and the size, direction and number of knurls materially differ. A practical expert from the factory where these bullets were made declared that they were alike, and not until his attention was directed to the canneluring and knurling did he perceive their differences.

Figure 7 is an enlargement of what was known as the "mortal" bullet in a recent trial for murder. The bullet was badly bruised and disfigured, but the rifling of the weapon had left some markings which conclusively proved that the defendant's pistol was the one from which it had been fired and the number and direction of its knurls, with the width and depth of its cannelure, showed it to be like the bullet found in the cartridge, shown in the left field of Fig. 6, a number of which were found in connection with the case, which belonged to the defendant. The bullets like the "mortal" bullet were 1100-1000ths of an inch in circumference and their cannelures had 42 knurls which were slightly obliqued from left to right, as shown in the illustration. The groove of the weapon rifling and that of the land upon the bullet each measured 105-1000ths of an inch. By computation, it was shown that the land upon the bullet should contain four knurls, and that each knurl should cover 26.2 thousandths of an inch. Inspection of the bullet in question disclosed four knurls within the space, or width of each of its lands, which were present only in part. Other peculiarities, discovered by microscopic examination, clearly completed the identification of the missile, and established the fact, that it was like the other bullets found in defendant's possession. The details in Fig. 7 are not so well shown as they might be, but it is the only thing of the kind, now at my command.

The subject which I have presented, is one which for its proper elucidation requires vastly more than my allotted time, and I leave it with a dissatisfied feeling of my incompetency to deal with it, as successfully as might be done under different circumstances.

## Figures and Tables

**Figure f1:**
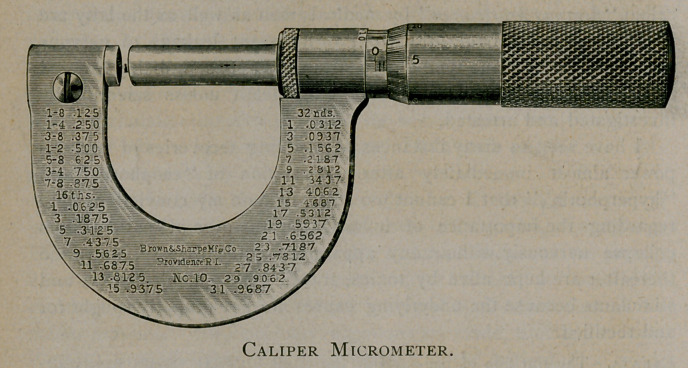


**Figure f2:**
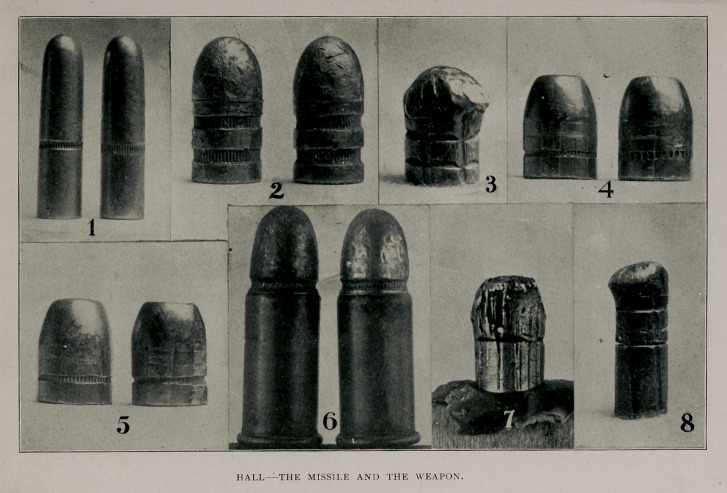


**Figure f3:**